# Association between serum vitamin C and HPV infection in American women: a cross-sectional study

**DOI:** 10.1186/s12905-022-01993-7

**Published:** 2022-10-05

**Authors:** Chunqin Zheng, Zhixiang Zheng, Weiqiang Chen

**Affiliations:** 1 Department of Obstetrics and Gynecology, Shantou Central Hospital, Shantou, 515000 China; 2 Department of Anesthesiology, Shantou Central Hospital, Shantou, 515000 China

**Keywords:** Vitamin C, Human papillomavirus, HPV, NHANES

## Abstract

**Background:**

Evidence regarding the relationship between serum vitamin C levels and human papillomavirus (HPV) infection is limited. Therefore, this study aimed to investigate whether serum vitamin C levels are independently associated with HPV infection.

**Methods:**

Data for this cross-sectional study were obtained from the National Health and Nutrition Examination Survey 2003–2006. A total of 2174 women, 18–59 years of age, were enrolled in this study. The associations between serum vitamin C levels (continuous and categorical forms) and cervicovaginal HPV infection were estimated using weighted logistic regression.

**Results:**

The adjusted binary logistic regression showed that serum vitamin C was not associated with the risk of HPV infection after adjusting for age, race, poverty income ratio, alcohol consumption, smoking, body mass index, education, and health condition (odds ratio [OR] 0.998, 95% confidence interval [CI] 0.994–1.001). Serum vitamin C levels were converted from a continuous variable to a categorical variable for the analysis. Compared with the vitamin C deficiency and hypovitaminosis groups, there was a negative correlation between vitamin C and HPV infection when vitamin C was adequate (OR 0.7, 95% CI: 0.52–0.94); however, when the serum vitamin C level was inadequate and saturated, this negative correlation was weaker or nonexistent (OR 0.76, 95% CI 0.56–1.03 and OR 0.76, 95% CI 0.55–1.04, respectively). A nonlinear relationship was detected between vitamin C level and HPV infection. Furthermore, we performed subgroup analysis of different models and found that serum vitamin C concentration was negatively associated with HPV infection in women ≥ 25 years of age; however, in women < 25 years of age, serum vitamin C levels were not associated with HPV infection.

**Conclusion:**

The results from this United States nationally representative sample supported the hypothesis that there was a U-shaped relationship between serum vitamin C levels and HPV infection. Future studies are warranted to assess the association between vitamin C and HPV persistence and clarify the underlying mechanisms of these associations.

## Background

Human papillomavirus (HPV) infection is a major public health challenge for women. According to a nationwide survey, 39.9 million United States (US) women (42.5%) aged 14–59 years were estimated to have at least one type of HPV infection in 2003–2006 [1]. While the majority of HPV infections are spontaneously cleared by natural immune responses, a portion of HPV infections become persistent, which can lead to disease [2]. Persistent HPV infection is an important sexually transmitted disease (STD), associated with more than 5% of all cancers worldwide [3]. HPV, especially variants 16 and 18, has been identified as the causative agent in the pathogenesis of cervical cancer [4], which continues to be among the top gynecologic cancers worldwide. According to the current data, it is ranked fourteenth among all cancers and fourth among cancers in women worldwide [5]. Globally, there were approximately 570 000 cases of cervical cancer and 311 000 deaths in 2018, demonstrating its significant global burden [6]. Additionally, HPV types 6 and 11 are detected in 90% of condyloma acuminata (CA) and warts. CA or warts is one of the most common STDs in the world [7]. HPV also causes cancer of the anus, vulva, and penis, and genital warts [8]. Three highly successful HPV vaccines have been introduced worldwide since 2006, and are projected to be on the national vaccine schedule of more than 150 countries by the end of 2021 [9]. The 9vHPV vaccine prevents approximately 90% of cervical cancers and HPV-related diseases.[10, 11]; however, several challenges remain [12]. Therefore, it is important to determine how to prevent HPV infection in common means.

Vitamin C (VC) is an essential and controversial nutrient in human physiology and pathology. It is a water-soluble vitamin, known chemically as l-ascorbic acid. Humans are unable to synthesize VC; therefore, they can only obtain it through food or tablets [13]. Both natural and synthetic ascorbic acid are chemically identical, and there are no known differences in their biological activities or bio-availability [13].

VC exerts a powerful antioxidant effect and is one of the most important natural nutrients. Additionally, it is required in many physiological events, including redox homeostasis, neuropeptide and collagen synthesis, histone demethylation, proteoglycan degradation, and regulation of hypoxia-inducible transcription factors [14, 15]. Moreover, one study showed that VC plays a role in preventing, shortening, and alleviating diverse infections [16]. Although the physiological role of VC is clear, its link to pathology, especially in complex diseases such as cancer, remains a matter of debate [17].

A case–control study by Giuliano et al. found that dietary intake of VC was negatively associated with the risk of HPV persistence [18]. Additionally, Barchitta et al. observed low dietary intake of VC in women with high-risk HPV infection [19]. Moreover, another study showed that VC intake has an inverse, dose-dependent association with the risk of cervical neoplasia [20]. Previous studies have examined the relationship between dietary VC, HPV infection, and cervical neoplasia. Although Basu et al. studied the association between plasma VC and HPV infection[21], the sample size was very small. No study has examined the relationship between serum VC levels and HPV infection in large samples.

Considering the relationship between VC and other infections, we postulated that serum VC might play a protective role against cervicovaginal HPV infection. The objective of the current study was to explore the association between serum VC levels and the prevalence of cervicovaginal HPV infection.

## Methods

### Data sources and study population

This cross-sectional study was restricted to women aged 18–59 years who completed HPV tests in the National Health and Nutrition Examination Survey (NHANES) from 2003 to 2006. Responses coded as “don’t know”, “refused,” “inadequate” or “missing” in the original NHANES data were treated as missing. Participants with missing HPV data, covariates, or VC levels were excluded. The NHANES is a nationally representative health survey in the US designed and administered by the National Center for Health Statistics (NCHS) at the Centers for Disease Control and Prevention (CDC). It is an ongoing survey that uses a complex multistage sampling design to obtain a representative sample of the US population during each collection cycle. It collected information on demographic indicators and health outcomes through interviews, face-to-face examinations, and laboratory tests. Each year, the NHANES examines approximately 5,000 participants per round, with participants located in different counties in the US. A computerized process randomly selects some, all, or no household members. Complete details regarding the NHANES study design, recruitment, procedures, and demographic characteristics can be accessed through the CDC website (https://www.cdc.gov/nchs/nhanes/index.htm). Briefly, NHANES study sampling consisted of a four-stage design with oversampling of some subgroups to improve precision. The NCHS ethics review board has approved the NHANES protocol. Written informed consent was obtained from all participants. The original study protocol was accessible on the website of the Ethics Review Board of the NCHS (https://www.cdc.gov/nchs/nhanes/irba98.htm) and was approved by the Ethical Review Committee (Protocol #98–12 and Protocol #2005–06). Furthermore, the NHANES covers interviews and medical examinations with a focus on various health and nutrition measurements, and is the main program of the NCHS. More detailed information can be found in the official NHANES website (https://www.cdc.gov/nchs/nhanes/).

### Measurement and classification of VC

Serum VC was collected and measured using isocratic high-performance liquid chromatography (HPLC) with electrochemical detection at 650 mV. Peak area quantification was based on a standard curve generated from three different concentrations of an external standard (0.025, 0.150, and 0.500 mg/dL). The quality assurance and quality control protocols utilized by the NHANES met the 1988 Clinical Laboratory Improvement Act mandate. Serum VC levels were modeled and analyzed in continuous and categorical forms. We categorized serum VC levels according to a prior study [22], as follows: deficiency and hypovitaminosis (0–23.99 µmol/L), inadequate (24–49.99 µmol/L), adequate (50–69.99 µmol/L), and saturating (≥ 70 µmol/L) based on participant plasma levels.

### Detection and classification of HPV infection

HPV infection was measured based on HPV genotyping using deoxyribonucleic acid (DNA) extracted from self-collected vaginal swabs. The DNA extracts used for the linear array HPV test were stored at  −20 °C for temporary storage and at −80 °Cfor long-term storage. The NHANES performed Roche Linear Array HPV genotyping tests for self-collected vaginal swab specimens and reported the results of HPV DNA detection tests for 37 HPV types. The HPV polymerase chain reaction summary variable indicates that if at least one HPV type is positive, the sample is negative. More information on HPV measurements can be found on the website (https://wwwn.cdc.gov/Nchs/Nhanes/2003-2004/L37SWA_C.htm#LBDHPCR).

### Covariates

The present study considered age, race/ethnicity, education, marital status, poverty income ratio (PIR), health condition, health insurance, smoking status, alcohol consumption, first age, body mass index (BMI), and levels of serum folate, albumin, *α*-carotene, and vitamin A, E, and D. Age was considered a continuous variable (18– 59 years). Participants self-reported race/ethnicity and were divided into five categories: Mexican American, other Hispanics, non-Hispanic white, non-Hispanic black, and other races. Education was categorized as high school graduate or lower, some college, and college graduate or above [23]. Marital status was recorded as married or living with a partner, never married, and widowed, divorced, or separated. PIR, the ratio of family income to the poverty threshold, ranged from zero to five. Participants’ self-reported health condition was classified into two categories: poor and fair were referred to as “poor”; good, very good and excellent were referred to as “good”. Participants reported their health insurance coverage (‘yes’ or ‘no’) from any source (e.g., private individual insurance, employer provided, Medicare, Medicaid, and Veteran’s Administration). Smokers were defined in the questionnaire as those who smoked more than 100 cigarettes per day. Consumption of at least 12 alcoholic beverages in any year was defined as alcohol consumption. The first age was defined as the age when the participants first had vaginal, anal, or oral sex. The number of partners was defined as the number of males with whom the participants have had vaginal, anal, or oral sex with in their lifetime. BMI was calculated for all participants by dividing the weight (kg) by the squared height (m^2^). The laboratory data included serum folate (nmol/L), albumin (g/L), α-carotene (µmol/L), vitamin A (µmol/L), vitamin E (µmol/L), and vitamin D (nmol/L) levels.

### Statistical analysis

All analyses were performed using the statistical software package R-4.0.2 (http://www.R-project.org, The R Foundation) and Free Statistics software version 1.7. We used the Medical Examination Center examination sampling weights provided by the NCHS to account for the unequal probability of selection and non-response. All estimates shown were weighted using these sampling weights, except when reporting the sample size by demographic characteristics. Descriptive statistics (sample sizes and weighted proportions) were computed along with mean serum VC levels and weighted prevalence of categorical serum VC levels. We estimated the crude odds ratios (ORs) and 95% confidence intervals (CIs) between serum VC levels and HPV infection using weighted logistic regression. Baseline characteristics were analyzed using means, standard errors (SE), percentages, or frequencies. Continuous variables were compared using analysis of variance for normally distributed variables and non-parametric tests for non-conformity to normal distribution. Categorical variables were analyzed using the chi-squared test. We adjusted the p-values of the multiple tests for a large number of tests using Bonferroni correction. The effect of VC on HPV infection was evaluated using multiple logistic regression models as follows: Model I: No adjustment; Model II: Adjusted for age, race/ethnicity, PIR, alcohol, smoking, BMI, education, and health condition; Model III: Adjusted for the variables in Model II plus first age and partner number; Model IV: Adjusted for the variables in Model III plus vitamin A level, health insurance, and marital status. Additionally, age was divided into two groups (< 25 years and ≥ to 25 years), and subgroup analysis was performed. Statistical significance was set at *P* < 0.05.

## Results

### Baseline characteristics of selected participants

**Fig. 1:**
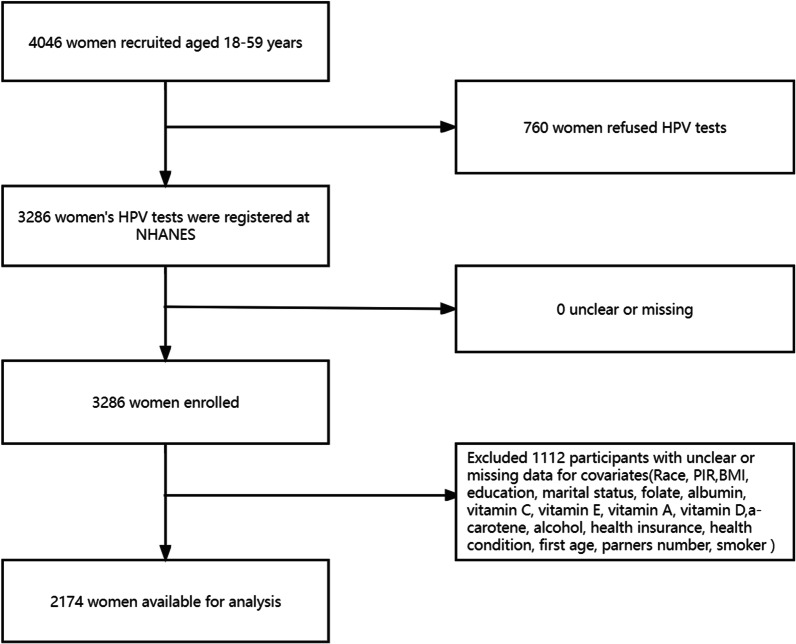
Flowchart of patient selection

Between 2003 and 2006, 4046 women aged 18–59 years were registered in the NHANES database; among them, 760 women refused to undergo HPV testing. Additionally, 1112 women’s covariates were unclear or missing; therefore, 2174 were included in our final analysis. More details of the selected sample are shown in the flowchart (Fig. 1). The baseline characteristics of the selected women according to HPV infection (dichotomous variable) are presented in Table 1.

**Table 1:** Baseline characteristics of participants

	Total Participants		HPV infection		No HPV infection		
Variables	(*n* = 2174)		(*n* = 1011)		(*n* = 1163)		*p*-value
	No. (%)	SE	No. (%)	SE	No. (%)	SE	
Age, mean, *y*	39.9	0.3	38.7	0.4	40.9	0.4	0.002
Race/Ethnicity							0.011
Mexican American	423 (7.5)	1.1	183 (7.5)	1.3	240 (7.4)	1	
Other Hispanic	70 ( 3.6)	0.6	36 (4.0)	0.7	34 (3.3)	0.8	
Non-Hispanic White	1100 (72.2)	2.4	456 (67.4)	2.7	644 (75.8)	2.5	
Non-Hispanic Black	492 (11.9)	1.5	301 (16.7)	2	191 (8.2)	1.2	
Other Race	89 ( 4.8)	0.7	35 ( 4.3)	0.8	54 (5.3)	1	
PIR, mean	3.1	0.1	2.8	0.1	3.4	0.1	< 0.001
Folate, mean, nmol/L	30.8	0.8	28.6	0.7	32.6	1.2	< 0.001
Albumin, mean, g/L	41.5	0.1	41.2	0.1	41.7	0.1	0.004
Vitamin C, mean,umol/L	55.8	1.2	52.8	1.5	58	1.3	0.002
Vitamin E, mean, umol/L	28.7	0.4	28.3	0.5	29	0.4	0.003
A.Carotene, mean, umol/L	0.1	0	0.1	0	0.1	0	< 0.001
Vitamin A, mean, umol/L	1.9	0	1.9	0	1.9	0	0.916
Vitamin D, mean, nmol/L	62.8	1.2	61	1.3	64.2	1.3	0.012
BMI, mean, kg/m^2^	28.4	0.2	28.5	0.3	28.3	0.3	0.616
Alcohol							0.003
Yes	1398 (70.5)	1.6	678 (74.8)	1.7	720 (67.3)	2.1	
No	776 (29.5)	1.6	333 (25.2)	1.7	443 (32.7)	2.1	
Health insurance							0.013
Yes	1698 (82.2)	1.5	762 (79.2)	1.9	936 (84.5)	1.7	
No	476 (17.8)	1.5	249 (20.8)	1.9	227 (15.5)	1.7	
First age, mean, y	17.6	0.1	17.2	0.1	18	0.1	< 0.001
Partner number, mean	10.2	1.7	10.8	0.6	9.7	2.9	< 0.001
Smoking status							0.001
Yes	877 (43.9)	1.1	456 (50.0)	2.2	421 (39.3)	1.5	
No	1297 (56.1)	1.1	555 (50.0)	2.2	742 (60.7)	1.5	
Education							< 0.001
High school graduate or less	892 (34.4)	1.4	463 (39.1)	1.9	429 (30.7)	1.7	
Some College	766 (37.0)	1.2	357 (36.3)	1.5	409 (37.6)	1.8	
College Graduate or above	516 (28.6)	1.9	191 (24.6)	2.1	325 (31.7)	2.3	
Marital status							< 0.001
Married or living with partner	1404 (67.5)	1.4	545 (56.4)	1.9	859 (75.9)	1.6	
Widowed, divorced, or separated	373 (17.1)	1.2	222 (22.6)	1.9	151 (13)	1.4	
Never married	397 (15.4)	1.2	244 (21.0)	1.6	153 (11.1)	1.3	
Health condition							0.054
Good	1789 (86.0)	0.9	813 (84.2)	1.4	976 (87.4)	1	
Poor	385 (14.0)	0.9	198 (15.8)	1.4	187 (12.6)	1	
Vitamin C (umol/L)							0.003
^23.99	271 (14.8)	1.4	151 (18.4)	1.8	120 (12.0)	1.5	
≤24–49.99	576 (25.0)	1.3	279 (25.0)	1.7	297 (25.0)	1.4	
50–69.99	749 (32.6)	1.8	330 (31.7)	1.8	419 (33.3)	2.2	
≥70	578 (27.6)	1.7	251 (24.9)	2	327 (29.7)	2.1	

*PIR* Poverty income ratio, *BMI* Body mass index, OR Odds ratio, *CI *Confidence interval

### The association between VC and HPV infection

In this study, we constructed four main models to explore the independent effects of VC on HPV infection using univariate and multivariate binary logistic regressions. The effect sizes (OR) 95% CI and P values are listed in Table 2. We found that the serum VC level was negatively associated with the risk of HPV infection in the non-adjusted model, even though serum VC level was converted from a continuous variable to a categorical variable for analysis.

**Table 2:** Association between Vitamin C and HPV in multiple regression

Variable	Model I		Model II		Model III		Model IV	
	OR(95% CI)	*f*-value	OR(95% CI)	*f*-value	OR(95% CI)	*f*-value	OR(95% CI)	*f*-value
Vitamin C	0.995 (0.991 ~ 0.998)	0.001	0.998 (0.994 ~ 1.001)	0.183	0.998 (0.994 ~ 1.001)	0.222	0.998 (0.994 ~ 1.001)	0.24
Vitamin C (umol/L)								
≤23.99	1(Ref)		1(Ref)		(Ref)		(Ref)	
24–49.99	0.75 (0.56–1)	0.048	0.76 (0.56–1.03)	0.076	0.75 (0.55–1.02)	0.067	0.76 (0.56–1.04)	0.082
50–69.99	0.63 (0.47–0.83)	0.001	0.7 (0.52–0.94)	0.019	0.7 (0.52–0.95)	0.022	0.71 (0.52–0.96)	0.026
≥70	0.61 (0.46–0.82)	0.001	0.76 (0.55–1.04)	0.083	0.76 (0.56–1.05)	0.094	0.77 (0.56–1.06)	0.113
Trend.Test	0.86 (0.79–0.94)	0.001	0.93 (0.85–1.02)	0.143	0.94 (0.85–1.03)	0.179	0.94 (0.85–1.03)	0.202

Mode I: Non-adjusted

Model II: Adjust for age, race/ethnicity, PIR, alcohol, smoker,BMI, education and health condition

Model III: Adjust for the variables in Model II plus first age and partner number

Model IV: Adjust for the variables in Model III plus vitamin A, health insurance, marital status

Using prior literature and a 10% change in the estimation method, we adjusted for variables that might potentially confound the association between serum VC levels and cervicovaginal HPV infection. We found that the relationship between VC levels and HPV infection was nonlinear. As presented in Table 2 (Model I), compared with the VC deficiency and hypovitaminosis groups, the inadequate and the saturating groups showed an OR of 0.76 (95% CI 0.56–1.03) and 0.76 (95% CI 0.55–1.04), respectively; however, compared with the deficiency and hypovitaminosis groups, the adequate group showed an OR of 0.7 (95% CI 0.52–0.94). As presented in Table 2 (Model II), compared with the VC deficiency and hypovitaminosis groups, the inadequate and the saturating groups showed an OR of 0.75 (95% CI 0.55–1.02) and 0.76 (95% CI 0.56–1.05), respectively; however, compared to the deficiency and hypovitaminosis group, the adequate group showed an OR of 0.7 (95% CI 0.52–0.95). Compared with Model II, the inclusion of vitamin A level, health insurance, and marital status in the same model (Model III) showed similar trends of associations between both models.

**Fig. 2:**
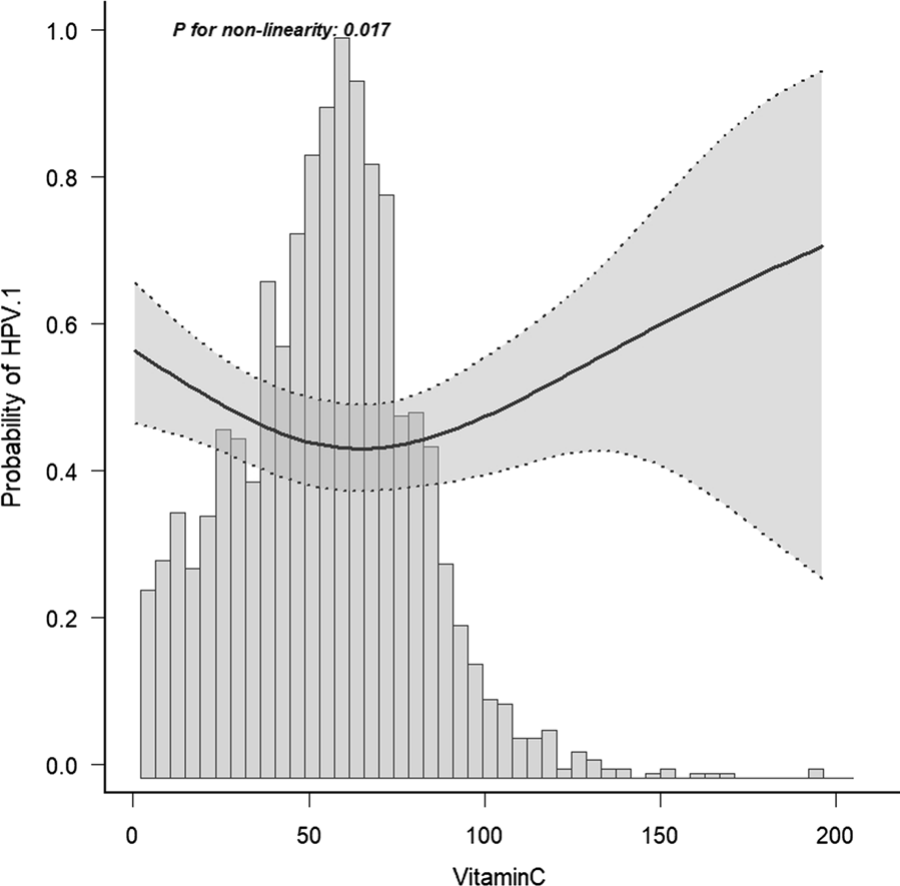
Adjusted for age, race, PIR, alcohol, smoker, BMI, education and health condition

In our study, we aimed to analyze the nonlinear relationship between serum VC levels and HPV infection (Fig. 2 and Table 3). We found that the relationship between VC and HPV infection was nonlinear after adjusting for age, race/ethnicity, PIR, alcohol consumption, smoking, BMI, education, and health condition using a smooth curve. Both binary logistic regression and two-piecewise binary logistic regressions were used to fit the relationship and choose the best-fit model based on P for the likelihood ratio test. Two-piecewise binary logistic regression was used to fit the association between serum VC levels and HPV infection because it can accurately represent this relationship. Using two-piecewise binary logistic regression and a recursive algorithm, the inflection point was calculated as 69.5. On the left side of the inflection point, the effect value and 95% CI were 0.9936 and 0.9878–0.9996, respectively; on the right, the effect size and 95% CI were 1.0102 and 1–1.0205, respectively.

**Table 3:** The results of the two-piecewise linear model

Outcome	HPV infection (OR, 95%CI)	*P*-value
Fitting model by two-piecewise linear regression		
Inflection point of vitamin C, umol/L	69.5	
< 69.5	0.9936 (0.9878– 0.9996)	0.0353
> 69.5	1.0102 (1– 1.0205)	0.04939
Likelihood ratio test		0.009

Adjust for age, race, PIR, alcohol, smoker, BMI, education, health condition

**Fig. 3:**
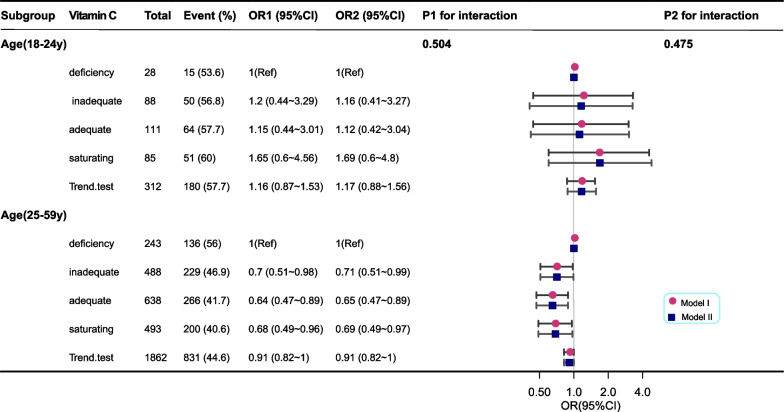
Model I: Adjusted for age, race/ethnicity, PIR, alcohol, smoker, BMI, education and health condition, first age and partner number. Model II: Adjusted for the variables in Model I plus vitamin A level, health insurance, and marital status

Furthermore, we performed subgroup analysis of different models (Fig. 3). Model I was adjusted for age, race/ethnicity, PIR, alcohol, smoking, BMI, education and health condition, first age, and partner number. Model II adjusted the variables in Model I and included vitamin A level, health insurance, and marital status. Serum VC concentration was negatively associated with HPV infection in women ≥ 25 years of age; however, in women < 25 years of age, serum VC levels were not associated with HPV infection. This may be related to the high prevalence of HPV infection in women younger than 25 years and the high rate of auto-clearance after HPV infection.

## Discussion

We analyzed the prevalence of HPV in a subgroup of sexually active women in the US before the introduction of the HPV vaccine. Briefly, we found that serum VC level, as a continuous variable, was not associated with HPV infection after adjusting for other covariates (OR 0.998; 95% CI 0.994–1.001). It seems that, by making VC a categorical variable, a nonlinear relationship is possible. Therefore, we further considered this nonlinear relationship and found a curvilinear relationship between serum VC levels and HPV infection. The risk of HPV infection was lowest when the VC was approximately 69.5 µmol/L. Patients with serum VC levels above or below 69.5 µmol/L had an increased risk. These results suggested an approximate U-shaped curve for the independent relationship between VC and HPV infection.

Our study analyzed subgroups according to age. In women aged < 25 years, serum VC levels were not associated with HPV infection; however, among US women aged ≥ 25 years, serum VC levels were negatively associated with HPV infection. To our knowledge, this is the first time that an association between serum VC and HPV infection has been observed in a large cross-sectional study of US women of different ages.

A study confirmed the association between inadequate VC levels and both acute and chronic inflammation [22]. Although the association between serum VC and HPV infection has not been studied, other infections have been reported to be correlated with VC. Carr et al. showed that supplementation with VC appears to be able to both prevent and treat respiratory and systemic infections [24]. Garaiova et al. performed a randomized controlled trial that found that 6 months of daily supplementation with the Lab4 probiotic and VC combination reduced the incidence of coughing, absenteeism, and antibiotic use in 3- to 10-year-old children [25]. In addition, there is strong evidence that VC can shorten the duration of respiratory virus infections [26–29].

There are different opinions on optimal VC levels for health and the classification of sufficient versus nonsufficient vitamin C levels; however, ≤ 23.99 µmol/L of serum VC is generally considered as a deficiency. Hypovitaminosis and VC deficiency causes scurvy. Given our findings, when the value of VC was around 69.5 µmol/L, the risk of HPV infection was the lowest. Unfortunately, a significant percentage of the US population have suboptimal serum VC levels. In our analysis, 14.8% of the women had a serum VC level of ≤ 23.99 µmol/L, and 39.8% had a serum level of ≤ 49.99 µmol/L. Since humans cannot synthesize VC, it must be supplied through diet, particularly vegetables and fruits, such as oranges, lemons, grapefruit, tomatoes, and broccoli. The US guidelines recommend a daily intake of VC of 90 mg for males and 75 mg for females over the age of 19 [30].

Naidu et al. performed a case–control study including 120 women with cervical cancer and 30 controls and showed that the concentration of VC may be significantly reduced in patients with cervical cancer compared to healthy individuals [31]. Barchitta et al. performed a cross-sectional study of 251 Italian women with normal cervical cytology and found a lower intake of VC in HPV-positive women [19]. Additionally, a case–control study by Giuliano et al. found that dietary intake of VC was negatively associated with the risk of HPV persistence [18]. These findings are partially consistent with our finding of a negative association between VC intake and HPV infection. However, unlike them, we found that this negative association was only present in women aged ≥ 25 years, and that the association between VC and HPV infection was absent in women younger than 25 years. We speculate that the reasons for the different results may be as follows: (1) regression analysis with multiple models and subgroup analysis with age grouping were not performed in previous studies; (2) different concentrations of VC may or may not produce different effects (i.e., antioxidative or pro-oxidative) [32]; (3) most of the previous studies focused on the association between dietary VC and HPV infection; and (4) the sample sizes of these studies were slightly smaller than that in our work.

Our study had the following advantages. First, our sample size was relatively larger compared with those of previous similar studies. Second, as this was an observational study that was susceptible to potential confounding factors, we used logistic regression analysis with multiple models and subgroup analysis with age grouping to minimize residual confounding factors. Third, to ensure the robustness of the data analysis and explore the true relationship between serum VC levels and HPV infection, we performed a series of sensitivity analyses to fully explain the nonlinear relationship. Fourth, VC is not a dietary vitamin. It was measured by isocratic HPLC with electrochemical detection at 650 mV, which avoids recall and measurement biases. Few studies have been conducted in recent years to elucidate the relationship between serum VC levels and HPV infection in different age groups. Furthermore, according to multiple sampling, the mean and SE representations were applied in our study for the population description, and the mean plus standard deviation was used in previous studies for the population description.

This study has some limitations. First, current HPV DNA detection methods for large epidemiological studies cannot distinguish whether HPV is from the participant or their partner, or whether it indicates active infection [33]. Second, the cross-sectional design did not allow us to infer temporality. Third, because the study participants were restricted to 2174 American women aged 18–59 years, it cannot be generalized to men or people beyond this age range. It is necessary to consider this aspect when extrapolating results to other populations. Given these limitations, well-designed multicenter controlled trials are essential to verify our findings.

## Conclusions

The results from this US nationally representative sample supported the hypothesis that there is a U-shaped relationship between serum VC levels and HPV infection. There was a negative correlation between the VC levels and HPV infection; however, we found that this negative correlation exists only when the age is between 25 and 59 years, and VC is adequate. When there is serum VC deficiency, hypovitaminosis, or when the VC is inadequate, and saturating, this negative correlation is weaker or nonexistent. Future studies are warranted to assess the association between VC and HPV persistence and clarify the underlying mechanisms of these associations.

The results of this study have clinical value as we have used data from NHANES to assess the association of serum VC levels with HPV infection. Our findings may contribute to further studies on its pathogenesis and to the literature.

## Data Availability

All data generated or analyzed during the current study are included in this published article. Any further inquiries can be directed to the corresponding author.

## Abbreviations

CA: Condyloma acuminata
CDC: Center for disease control and prevention
CI: Confidence interval
DNA: Deoxyribonucleic acid
HPLC: High-performance liquid chromatography
HPV: Human papillomavirus
NCHS: National center for health statistics
NHANES: National health and nutrition examination survey
OR: Odds ratio
SE: Standard errors
STD: Sexually transmitted disease
US: United States
VC: Vitamin C
